# Cytokines in sepsis: a critical review of the literature on systemic inflammation and multiple organ dysfunction

**DOI:** 10.3389/fimmu.2025.1682306

**Published:** 2025-11-05

**Authors:** Roberta Vella, Diego Panci, Francesco Carini, Ginevra Malta, Salvatore Vieni, Sabrina David, Giuseppe Davide Albano, Maria Puntarello, Stefania Zerbo, Antonina Argo

**Affiliations:** ^1^ Department of Precision Medicine in Medical, Surgical and Critical Area (MePreCC), University of Palermo, Palermo, Italy; ^2^ Department of Health Promotion, Mother and Child Care, Internal Medicine and Medical Specialties, Section of Legal Medicine, University of Palermo, Palermo, Italy; ^3^ Department of Biomedicine, Neurosciences and Advanced Diagnostics (BiND), University of Palermo, Palermo, Italy

**Keywords:** sepsis1, cytokines2, inflammation3, multiple organ dysfunction4, immunity5

## Abstract

Sepsis constitutes a profoundly heterogeneous and dynamic clinical syndrome, precipitated by a maladaptive host response to infection in which the immune system’s regulatory balance is fundamentally disrupted. The intricate interplay between proinflammatory and anti-inflammatory pathways, ordinarily responsible for maintaining immune homeostasis, becomes pathologically skewed. Within this altered immunological landscape, cytokines serve as pivotal mediators, orchestrating a cascade of cellular events that may culminate in a rapid transition from systemic hyperinflammation to a state of immune exhaustion or suppression. This review offers a critical synthesis of the current scientific literature on the immunopathogenesis of sepsis, with a particular emphasis on the molecular and cellular mechanisms governing cytokine regulation. Special attention is directed toward elucidating the contribution of these mediators to the onset and progression of multiorgan dysfunction syndrome (MODS), a central and often fatal complication of severe sepsis. Through an integrative examination of the principal immune signaling networks and pathophysiological processes involved in sepsis, this review provides a cohesive theoretical framework positioning immune dysregulation as the fundamental axis of clinical deterioration. Such an approach underscores the imperative for a deeper insight into the immunological architecture of sepsis, thereby laying the groundwork for the rational design of targeted, mechanism-based therapeutic strategies.

## 1 Introduction

Sepsis represents a multifactorial pathological condition defined by a profoundly dysregulated host immune response to infection, capable of precipitating a rapid and often fatal progression toward multiorgan dysfunction syndrome (MODS) (1). Central to the immune pathophysiology of sepsis is the aberrant production and activity of cytokines, biologically active molecules primarily involved in immune system modulation (2). Cytokines drive both the initial hyperinflammatory phase of sepsis and the subsequent immunosuppressive state (3).

In the septic context, the excessive and unregulated stimulation of innate immune system cells, particularly the pattern recognition receptors (PRRs), leads to a pathological surge in cytokine production, a phenomenon commonly referred to as a “cytokine storm” (4, 5). This state of sustained immune activation propagates oxidative stress, inflicting direct cellular and tissue injury, which ultimately leads to organ system dysfunction and clinical deterioration in sepsis (6–8).

The later stages of sepsis progression are characterized by a profound state of immunosuppression, commonly referred to as the compensatory anti-inflammatory response syndrome (CARS). This phase is characterized by extensive apoptosis of lymphocyte subsets, particularly T and B cells, alongside with the expansion of immunosuppressive populations and the secretion of anti-inflammatory mediators, notably interleukin-10 (IL-10) and transforming growth factor-beta (TGF-β). This immunological paralysis critically undermines the host’s capacity to mount effective responses against secondary infections and plays a pivotal role in the evolution of multiorgan dysfunction (6–10).

In parallel, cytokine dysregulation exerts a significant influence over cellular energy metabolism and endothelial integrity. The resultant endothelial injury, compounded by increased vascular permeability and compromised tissue perfusion, contributes to widespread microcirculatory dysfunction. Organs with high metabolic demands—such as the heart, kidneys, liver, gastrointestinal tract, lungs, and brain—are particularly susceptible to both structural and functional injury under the cumulative burden of aberrant cytokine signaling, inflammatory effector molecules, and oxidative stress (11–13).

A mechanistic understanding of cytokine dynamics—encompassing both the hyperinflammatory and immunosuppressive trajectories—is essential for the rational development of immunomodulatory therapeutic strategies (14). This review aims to critically examine the multifaceted roles of cytokines in the pathogenesis of sepsis, with a particular focus on the molecular underpinnings of cytokine storm phenomena, the development of immune paralysis, and their systemic effects on physiological homeostasis and organ function.

## 2 Sepsis definition

### 2.1 Definition and clinical criteria

Over the past five decades, the definition of sepsis has evolved gradually, reflecting an in-depth understanding of the mechanisms involved and an underscoring focus on clinical signs of organ dysfunction rather than signs of inflammation (15). Traditionally, sepsis has been conceptualized as a manifestation of systemic inflammatory response syndrome (SIRS), arising in the context of extensive and severe tissue injury secondary to microbial infection (3). This perspective has evolved significantly, particularly following the Third International Consensus Definitions for Sepsis and Septic Shock (Sepsis-3) in 2016, which redefined sepsis as a “life-threatening organ dysfunction caused by a dysregulated host response to infection (16, 17). This newer definition emphasizes the disruption of immune homeostasis and the complex pathophysiological mechanisms underpinning the host’s maladaptive response to infectious insults (18). Besides, this definition highlights the critical distinction between uncomplicated infection and sepsis, which lies in the presence of organ dysfunction presenting with a constellation of signs, including hypothermia or hyperthermia, tachycardia, increased respiratory rate, and alterations in leukocyte count (18). In this context, clinical and laboratory scores, such as the SOFA and qSOFA, have emerged as diagnostic tools for the early identification of patients with sepsis, as well as for predicting their prognosis. Although the utility of these scores for screening was scaled down in the latest 2021 guidelines, the current recommended parameters for screening and diagnosing sepsis emphasize organ dysfunction and infection as the key features of sepsis pathogenesis and pathophysiology (19).

### 2.2 Biphasic immune trajectory

**Figure 1:**
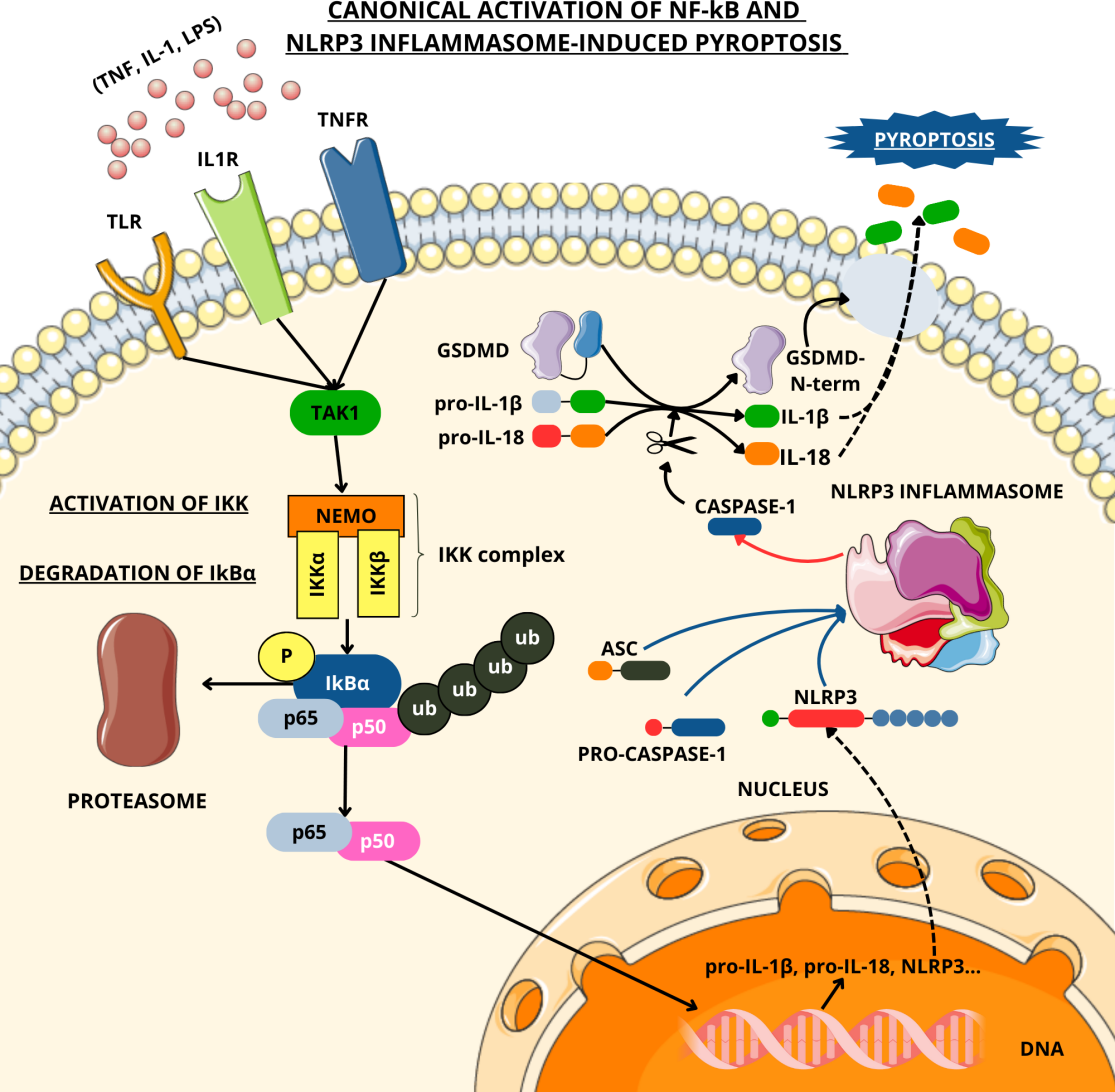
Canonical activation of NF-κB and NLRP3-induced pyroptosis. TLR, IL1R, and TNFR are activated, respectively, by LPS, IL-1, and TNF-α. These interactions trigger signaling cascades that involve various adaptor proteins and the kinase TAK1, resulting in the activation of the IKK complex, which phosphorylates the inhibitor IκBα, leading to its degradation via the proteasome. This allows the NF-κB subunits p65 and p50 to translocate into the nucleus, where they induce the transcription of immune-response genes. One of those genes encodes for NLRP3, which is the sensor component of the NLRP3 inflammasome, along with ASC and pro-caspase-1. Upon NLRP3 activation, pro-caspase-1 is cleaved into its active form, caspase-1. Caspase-1 processes the inactive forms of IL-1β and IL-18 (whose transcription has been activated via NF-κB) and cleaves gasdermin D (GSDMD). The N-terminal domain of GSDMD perforates the plasma membrane, inducing pyroptosis. The artwork used in this figure was adapted from Servier Medical Art (https://smart.servier.com/). Servier Medical Art by Servier is licensed under CC BY 4.0 (https://creativecommons.org/licenses/by/4.0/).

The development of SIRS is driven by the pathological surge in cytokine production (the “cytokine storm”) and its associated metabolic derangements, particularly those impacting oxidative metabolism balance. In sepsis, the innate immune system is activated through the recognition of pathogen-associated molecular patterns (PAMPs) and damage-associated molecular patterns (DAMPs) by PRRs. This molecular recognition induces a complex intracellular signaling cascade- primarily mediated through the nuclear factor kappa-light-chain-enhancer of activated B cells (NF-kb) pathway – that culminates in the transcriptional activation of proinflammatory cytokines, chemokines and antimicrobial peptides which ultimately leads to the development of SIRS (**Figure 1**). Inflammatory activation leads in turn to the recruitment and stimulation of innate immune cells, notably neutrophils and macrophages, that subsequently generate high levels of reactive nitrogen species (RNS). These reactive molecules exert cytotoxic effects, contributing to cellular injury and organ dysfunction (8).

Concurrently, recent advances in our understanding of sepsis pathogenesis have revealed a biphasic immune trajectory. Following the initial hyperinflammatory response, a compensatory and often deleterious immunosuppressive phase—termed the “compensatory anti-inflammatory response syndrome” (CARS)—emerges, leaving patients susceptible to opportunistic secondary infections and prolonged clinical deterioration (9).

## 3 Innate lmmunity and cytokine storm in sepsis

### 3.1 Pattern recognition receptors

Upon exposure to a pathogenic agent, the host’s immediate immunological defense is mediated by the innate immune system, a highly conserved network comprising both cellular and humoral components that function independently of prior antigenic experience to initiate and propagate the inflammatory response (4).

The activation of this system is initiated through the recognition of conserved microbial motifs, collectively termed pathogen-associated molecular patterns (PAMPs), by PRRs expressed on or within innate immune cells. PAMPs encompass a wide array of essential structural elements from invading microorganisms, including lipopolysaccharide (LPS), mannose residues, nucleic acids (DNA or RNA), flagellin, peptidoglycan, and lipoteichoic acid (LTA) (20).

In parallel, host cell injury—whether induced by infectious agents or by physical, chemical, or metabolic stressors—elicits the release of damage-associated molecular patterns (DAMPs), which are endogenous molecules of nuclear or cytosolic origin. These danger signals, by engaging the same PRR systems, further amplify the inflammatory response through a feed-forward mechanism that may escalate into systemic inflammation. Elevated serum levels of DAMPs have been correlated with adverse clinical outcomes in patients with sepsis, reflecting their role as prognostic biomarkers and potential mediators of disease severity (21, 22).

The signaling cascades initiated by PRRs are critical in orchestrating the subsequent phases of the immune response, particularly the recruitment and migration of leukocytes to sites of infection and tissue damage (23).

### 3.2 PRR families and functional roles

The PRRs encompass both membrane-bound and cytosolic proteins. Among the transmembrane group are the Toll-like receptors (TLRs) and C-type lectin receptors (CLRs). The TLRs are expressed by a wide range of immune and non-immune cell types, including neurons, which have been implicated in the amplification of cytokine storm phenomena. The cytosolic PRR families include the retinoic acid-inducible gene-I-like receptors (RLRs) and the nucleotide-binding oligomerization domain-like receptors (NLRs) (24–26). Clinical studies have demonstrated that individuals with sepsis exhibit elevated mRNA expression of TLR4, alongside with an upregulation of TLR2 receptor protein levels (4).

Upon engagement with pathogen- or damage-associated molecular patterns (PAMPs or DAMPs), PRRs expressed on the surface of monocytes and antigen-presenting cells (APCs) initiate intracellular phosphorylation cascades that culminate in a prototypical inflammatory response (5).

Except for specific NLR subtypes, this receptor-mediated sensing event induces transcriptional activation of a repertoire of genes involved in the inflammatory milieu, notably those encoding proinflammatory cytokines, chemotactic factors, and antimicrobial effectors (24).

The intracellular signaling pathways activated by PRR engagement converge on two key transcriptional regulators: interferon regulatory factors (IRFs), which control the expression of type I interferons, and nuclear factor kappa-light-chain-enhancer of activated B cells (NF-κB) (3). NF-κB signaling is activated across diverse tissues and cellular contexts, where it may exert protective effects in some environments while contributing to tissue damage and dysfunction in others (27).

## 4 Cytokine storm and sepsis

### 4.1 Definition and pathogenesis

The disruption of cytokine homeostasis and its associated signaling pathways initiates complex immunological cascades, often driven by uncontrolled feedback loops that amplify the response beyond physiological limits (5). This dysregulation can culminate in a pathological phenomenon known as the “cytokine storm” (CS), a term first introduced by Ferrara and colleagues in 1993 (28). Characterized by an excessive and disordered immune activation, CS can emerge in the context of sepsis and is associated with significant tissue injury and the potential evolution toward multiorgan dysfunction syndrome (MODS) (26).

### 4.2 Cytokines overview

The term “cytokines” was first introduced in 1974 by Stanley Cohen and colleagues to describe a class of biologically active molecules primarily involved in immune system modulation (29). These molecules are secreted by various cell types and have the capacity to influence the behavior of other cells. Depending on the physiological context, cytokines may exert their effects through autocrine, paracrine, or combined signaling mechanisms. Although their actions are typically confined to the local microenvironment, cytokines can also exert systemic effects, thereby demonstrating their functional versatility and central role in immune regulation and intercellular communication (30).

Cytokines are grouped into several major families based on structural and functional characteristics, including interleukins (ILs), interferons (IFNs), chemokines, tumor necrosis factors (TNFs), transforming growth factors (TGFs), and colony-stimulating factors (CSFs). Functionally, cytokines exhibit diverse functional profiles, with proinflammatory, anti-inflammatory, or dual immunomodulatory effects depending on the context (29–33) (**Table 1**).

**Table 1:** Most relevant cytokines in sepsis pathogenesis, with details on their main functions and key information.

Category	Cited members	Functions	Key notes
Interleukins (IL)	IL-1α, IL-1β, IL-18, IL-33, IL-36 (proinflammatory); IL-1RA, IL-4, IL-10, IL-37 (anti-inflammatory); IL-6 (dual); IL-3	Innate immunity activation, inflammation regulation	IL-6: dual behavior depending on receptor (proinflammatory via IL-6R, anti-inflammatory via IL-6Rα). IL-3: involved in cytokine storm regulation during sepsis.
Tumor Necrosis Factors (TNF)	TNF-α, LT-α, LT-β, FasL, CD40L, CD30L, CD27L, TRAIL/APO-2L	Modulation of cellular responses, inflammation, apoptosis	TNF-α: produced by monocytes/macrophages; activates NF-κB, JNK, ERK pathways; involved in autoimmune, cardiovascular, and inflammatory diseases.
Interferons (IFNs)	IFN-γ (Type II)	Antiviral defense, activation of innate and adaptive immunity	IFN-γ: activates macrophages; induces IL-6, TNF-α, IL-10 production via JAK-STAT; IFNγR1 deficiency increases infection susceptibility.
Complement System	CCL1, CCL2, CCL8, CCL20 (CC); CXCL8, CXCL10, CXCL12 (CXC)	Leukocyte migration, inflammation signaling	Key roles in sepsis severity and prognosis; CXCR2 regulates neutrophil recruitment; upregulated in cytokine storm and ARDS
Colony-Stimulating Factors (CSFs)	GM-CSF, G-CSF, M-CSF	Myeloid cell differentiation and activation	GM-CSF: promotes both pro- and anti-inflammatory pathways; modulates survival and function of various immune cells; impact depends on individual immune status.
Transforming Growth Factors (TGFs)	TGF-β1, β2, β3	Immunosuppression, cell proliferation and differentiation	Promotes Tregs with IL-2, or Th17 cells with IL-6/IL-21; excess TGF-β impairs early immune responses and suppresses T/B cells (except Tregs).

### 4.3 Pro- and anti-inflammatory cytokines involved

The onset of CS typically involves the rapid elevation of proinflammatory mediators such as interleukin-1 (IL-1) and tumor necrosis factor-alpha (TNF-α), resulting in a hyperinflammatory state. In parallel, compensatory mechanisms are activated to mitigate this inflammatory surge, including the upregulation of anti-inflammatory cytokines such as interleukin-10 (IL-10) and transforming growth factor-beta (TGF-β) (32).

#### 4.3.1 Associated immunosuppression

The excessive secretion of anti-inflammatory cytokines, extensive lymphocyte apoptosis through intrinsic and extrinsic pathways, proliferation of regulatory immune cell populations—including regulatory T cells and myeloid-derived suppressor cells—and the concurrent dysfunction or numerical depletion of macrophages, dendritic cells, and innate phagocytes, all contribute to the profound immunosuppressive state associated with sepsis. This immunological collapse severely compromises host defenses, facilitating opportunistic infections and perpetuating organ injury consistent with MODS (4, 10).

### 4.4 Interleukins

The interleukin-1 (IL-1) family comprises a group of prominent proinflammatory cytokines, which are further subclassified based on sequence homology and receptor specificity. This family includes 11 soluble ligands and 10 corresponding receptors. Key proinflammatory members include IL-1α, IL-1β, IL-18, IL-33, and IL-36. In contrast, certain members of this family serve regulatory functions; for instance, IL-37 exhibits potent anti-inflammatory activity (34).

Additional interleukins implicated in the proinflammatory response during sepsis include IL-6, IL-12, and IL-17, which play critical roles in immune cell activation and cytokine induction (9). IL-12 promotes the production of interferon-gamma (IFNγ) by T helper and natural killer (NK) cells (35). IL-17A and IL-17F, produced by various immune subsets in response to stimuli such as IL-1β and IL-23, significantly contribute to host defense mechanisms by enhancing neutrophil recruitment, stimulating antimicrobial peptide synthesis, and activating macrophages—particularly in synergy with IFN-γ. Moreover, IL-17 facilitates the release of additional proinflammatory cytokines and matrix metalloproteinases (MMPs), which support tissue repair but also contribute to sustained inflammation and potential tissue injury (36).

Prominent anti-inflammatory interleukins include IL-1 receptor antagonist (IL-1RA), IL-4, IL-10, and IL-37. IL-10 serves as a central immunoregulatory cytokine, suppressing the production of proinflammatory mediators such as TNF-α, inhibiting CD4+ T lymphocyte proliferation, and promoting the differentiation of regulatory T cells (Tregs), thereby fostering an immunosuppressive environment. IL-4 supports T helper 2 (Th2) cell differentiation, induces the expression of other anti-inflammatory cytokines, and concurrently downregulates proinflammatory pathways. IL-37 attenuates both innate and adaptive immune responses. Additionally, IL-3 has recently been implicated in modulating cytokine storm phenomena in sepsis, as it influences myelopoiesis and the corticosteroid response (4, 10).

Interleukin-6 (IL-6), traditionally considered a proinflammatory cytokine, is now recognized for its context-dependent dual functionality (37). The functional outcome of IL-6 signaling depends on the balance between classic signaling, mediated by membrane-bound IL-6 receptor and gp130, and trans-signaling, mediated by soluble IL-6 receptor and gp130 on virtually all cells. This balance does not remain static but shifts dynamically during sepsis. In the hyperinflammatory phase (cytokine storm), the increased shedding of membrane IL-6 receptor by metalloproteases such as ADAM17 and ADAM10 leads to the accumulation of soluble IL-6 receptor, thereby amplifying proinflammatory trans-signaling. This mechanism enhances endothelial activation and monocyte recruitment, sustaining the overwhelming inflammatory response. In contrast, during the compensatory anti-inflammatory response syndrome (CARS), a relative rise in regulatory pathways such as soluble gp130, which buffers IL-6/sIL-6R complexes, and intracellular regulators including STAT3 and SOCS3, shifts the balance toward classic anti- inflammatory signaling. At this stage, IL-6 signaling is primarily associated with tissue repair and anti-inflammatory activity, particularly in hepatocytes and epithelial cells. Experimental studies confirm that selective inhibition of trans-signaling with sgp130Fc improves survival in sepsis models by attenuating excessive inflammation without compromising the protective functions of classic signaling (38). These findings highlight that IL-6 acts as a stage-dependent mediator in sepsis, with proinflammatory trans-signaling dominating the acute phase and anti-inflammatory or regenerative classic signaling prevailing during immunosuppression (39, 40). IL-8 can play a dual role as well: it can promote proinflammatory cytokines such as IL-1β and TNF-α, but it can also inhibit the production of these cytokines in specific contexts, showing an anti-inflammatory effect and a regulatory role (32). Indeed, in addition to its well-established role as a neutrophil chemoattractant through CXCR1 and CXCR2, IL-8 (CXCL8) acts as a stage-dependent mediator in sepsis. During the early hyperinflammatory phase, IL-8 amplifies the inflammatory cascade by enhancing neutrophil activation, reactive oxygen species release, and secondary production of IL-1β and TNF-α. Conversely, under conditions of prolonged stimulation or high receptor occupancy, IL-8 can induce receptor desensitization and downregulate NF-κB activity, thereby attenuating the production of proinflammatory cytokines and contributing to immune regulation. Clinically, elevated circulating IL-8 levels are strongly associated with disease severity and mortality in septic patients. IL-8 has been validated as one of the biomarkers in the PERSEVERE panel for stratifying prognosis in pediatric septic patients (41). These findings underscore the dual role of IL-8 as both an inflammatory amplifier and a potential regulator in the immunopathogenesis of sepsis.

### 4.5 Tumor necrosis factors

Tumor necrosis factors (TNFs), a family of pleiotropic cytokines, are central to both homeostatic regulation and pathological inflammation (42). Key members include TNF-α, lymphotoxins α and β (LT-α, LT-β), Fas ligand (FasL), CD40L, CD30L, CD27L, and TRAIL/APO-2L (43). TNF-α, predominantly produced by monocytes and macrophages, exerts both proinflammatory and immunoregulatory effects (43, 44). Experimental models have shown that TNF-α deficiency impairs host immune competence, increasing susceptibility to infections. It induces apoptotic and necrotic cell death via receptor-mediated pathways, particularly through the activation of TNFR-1 (45). Additionally, TNF-α promotes B cell activation and is involved in the pathophysiology of various diseases, including cardiovascular, neurological, autoimmune, and metabolic ones. As a pyrogen, it activates multiple signaling cascades, including NF-κB, ERK, p38 MAPK, and JNK, regulating downstream expression of inflammatory mediators such as IL-6, IL-8, and IL-18 (46). In murine models, TNF-α becomes detectable within 30–90 minutes following exposure to lipopolysaccharide (LPS) and initiates subsequent inflammatory cascades. In septic patients, elevated TNF-α levels have been correlated with higher mortality rates (47). Given its pathogenic relevance, TNF signaling represents a prominent target for therapeutic intervention (46), despiteexperimental studies have yielded conflicting results on the clinical benefit of anti TNF-α agents (48).

### 4.6 Interferons

Interferons (IFNs), which are classified into three major types, orchestrate antiviral defense and immunomodulatory responses. Among these, interferon-gamma (IFN-γ) is the principal type II interferon (4) and is critical for both innate and adaptive immune responses. Its activity is mediated through the Janus kinase-signal transducer and activator of transcription (JAK-STAT) pathway following engagement with the IFN-γ receptor (IFNγR). IFN-γ enhances macrophage activation and induces downstream production of IL-6, TNF-α, and IL-10 (49). Genetic deficiencies in IFNγR1 compromise host immunity and increase vulnerability to infection (50) since some pathogens have developed mechanisms to inhibit this receptor as a means of immune evasion (51).

### 4.7 Chemokines

Chemokines, a diverse family of low-molecular-weight cytokines, signal through G protein-coupled receptors expressed on leukocytes and endothelial cells, mediating cellular recruitment and migration during immune responses (52, 53). These molecules are classified into four structural subfamilies—CXC, CC, CX3C, and C—based on the positioning of conserved cysteine residues (54). Chemokines have emerged as both biomarkers and potential therapeutic targets in sepsis. Elevated serum concentrations of several chemokines—including CCL1, CCL2, CCL8, CCL20, CXCL8, CXCL10, and CXCL12—have been associated with severe meningococcal sepsis and increased mortality in pediatric cohorts, reinforcing their prognostic significance (55). Receptors such as CXCR2 regulate neutrophil migration and inflammatory responses (55). In COVID-19-associated cytokine storms, heightened expression of CXCL10, CXCL8, and CCL2 has been implicated in the pathogenesis of acute respiratory distress syndrome (ARDS) (53).

### 4.8 Hematopoietic growth factors

Growth factors such as granulocyte-macrophage colony-stimulating factor (GM-CSF), macrophage colony-stimulating factor (M-CSF), and granulocyte colony-stimulating factor (G-CSF) also play pivotal roles in sepsis pathobiology by promoting myelopoiesis and modulating cytokine production (4). *In vitro* evidence indicates that GM-CSF exerts diverse effects on hematopoietic and myeloid cells, including regulation of survival, proliferation, differentiation, and activation of monocytes, neutrophils, eosinophils, and basophils. Its immunological impact includes both pro- and anti-inflammatory functions (56), promoting the expression of Th1-type cytokines while suppressing IL-10 and IL-4 (57). Although GM-CSF has been explored as a means to restore immune competence in sepsis, its early use may exacerbate hyperinflammation, whereas its deficiency may worsen immunoparesis, indicating that its therapeutic utility is likely context-dependent and influenced by individual immune profiles (9, 56, 57).

### 4.9 TGF-β and regulatory roles

Transforming growth factor-beta (TGF-β), which exists in three isoforms (β1, β2, β3) (58), is a potent immunoregulatory cytokine involved in cell proliferation and differentiation. It signals through TβRII and TβRI/ALK5 receptors, activating the canonical Smad2/3–Smad4 pathway, but also engages non-canonical cascades such as MAPK, PI3K/Akt, and Rho-like GTPases, thereby broadening its range of biological effects. It exerts a dual effect on T cell fate: in the presence of IL-6 or IL-21, it promotes Th17 differentiation, while in conjunction with IL-2, it induces Foxp3 expression, favoring regulatory T cell expansion (59). Overproduction of TGF-β impairs innate immune responses—such as interferon signaling, natural killer cell cytotoxicity, and macrophage activation—while simultaneously dampening adaptive immune functions, except for Treg cell maintenance, thereby contributing to the immunopathogenesis of sepsis (60). TGF-β also exerts opposing functions depending on the cytokine milieu. In the presence of interleukin-6, interleukin-21, and interleukin-23, TGF-β directs naïve CD4+ T cells towards the Th17 lineage through STAT3 activation, thereby promoting sustained inflammation. In contrast, when interleukin-2 dominates, TGF-β drives Foxp3-dependent T regulatory differentiation through Smad signaling, enhancing immunosuppression. In sepsis, the early surge of interleukin-6 favors Th17 expansion, while in later stages the predominance of T regulatory cells contributes to immune paralysis. Beyond shaping T cell polarization, TGF-β directly influences innate immune pathways: excessive signaling suppresses type I interferon responses, reduces natural killer cell cytotoxicity, and limits macrophage activation, weakening host defense.

### 4.10 Cytokines-based therapeutic strategies

The high mortality associated with sepsis reflects not only the limitations of current therapeutic approaches—antimicrobials, source control, and supportive care—but also the complexity of its underlying pathophysiology. Sepsis arises from a dysregulated immune response, characterized by an initial hyperinflammatory phase followed by a state of immunosuppression, both of which are insufficiently addressed by existing treatment modalities. In this context, immunomodulatory strategies—ranging from cytokine-directed therapies to interventions targeting intracellular inflammatory pathways—are increasingly recognized as promising approaches to reduce mortality and improve clinical outcomes.

Therapeutic strategies under investigation include the use of cytokines that enhance the viability and function of immune cells, such as interleukin-7 (IL-7). A substantial body of evidence indicates that sepsis is associated with marked lymphopenia, characterized by reductions in T cells, B cells, dendritic cells, and natural killer (NK) cells. This depletion is frequently accompanied by the involution of lymphoid organs, including the thymus, spleen, lymph nodes, and bone marrow—processes largely driven by apoptosis—and has been documented in both adult and neonatal populations with sepsis (6).

In this context, cytokine-targeted therapy may reverse sepsis-induced lymphopenia and improve lymphocyte function. A recent double-blind, randomized, placebo-controlled trial investigated the intravenous administration of CYT107, a recombinant human interleukin-7 (IL-7), in patients with septic shock and severe lymphopenia (61). The study reported that intravenous CYT107 effectively reversed sepsis-induced lymphopenia by significantly increasing absolute lymphocyte counts, with this effect persisting for several weeks after treatment. However, a considerable proportion of patients developed severe adverse events, most notably transient respiratory distress, which contributed to the premature termination of the trial.

Another crucial immunological target in sepsis is the pleiotropic cytokine interleukin-6 (IL-6), which plays a central role in organ dysfunction and sepsis-related mortality (62–64). Tocilizumab (TCZ), a humanized anti-IL-6 receptor antibody of the IgG1 subclass, acts by inhibiting the binding of IL-6 to both soluble and membrane-bound receptors, thereby reducing its pro-inflammatory activity (65). In clinical trials and off-label use, TCZ has demonstrated significant efficacy in conditions characterized by excessive IL-6 secretion (66). Its widespread use during the COVID-19 pandemic further underscores its therapeutic potential in hyperinflammatory states (67). However, the efficacy of TCZ in sepsis remains controversial and appears to be strongly influenced by the timing of administration, reflecting the complex and ambivalent role of IL-6 in sepsis pathophysiology.

The interleukin-1 receptor (IL-1R) represents another relevant therapeutic target in sepsis. Anakinra, a recombinant IL-1 receptor antagonist, has shown survival benefit in a specific subgroup of patients with MAS-like features, reducing 28-day mortality from 64.7% to 34.6% (68). In contrast, the PROVIDE trial reported early improvements in organ function and coagulation markers but no survival advantage at 28 days, likely due to premature treatment discontinuation while the hyperinflammatory state persisted (69). Overall, IL-1R blockade appears promising in selected hyperinflammatory phenotypes, although its impact on long-term survival remains uncertain and this might be due to the premature discontinuation of treatment.

These therapeutic avenues exemplify both the potential and the complexity of modulating intracellular signaling to temper the cytokine storm in sepsis and underscore the critical need for precise immunophenotypic stratification in sepsis trials (70).

## 5 Intracellular inflammation signaling pathways in sepsis

Within the pathophysiological framework of sepsis, the cytokine storm is underpinned by the activation of intracellular inflammatory signaling pathways, which assume a pivotal role in both the initiation and perpetuation of the dysregulated immune response. Pathways such as NF-κB, MAPKs, JAK-STAT, and the inflammasome complexes are engaged upon recognition of PAMPs and DAMPs by PRRs. Their activation leads to the transcriptional upregulation and secretion of pro-inflammatory mediators, including TNF-α, IL-1β, and IL-6, which in turn act in autocrine and paracrine loops to further amplify signaling cascades. This creates a self-sustaining cycle of cytokine release that propagates systemic inflammation, disrupts endothelial and metabolic homeostasis, and ultimately contributes to the organ dysfunction that defines sepsis. Thus, intracellular signaling pathways can be regarded not merely as passive transducers of microbial sensing, but as central drivers of the cytokine storm and, by extension, of the deleterious clinical manifestations of sepsis.

### 5.1 NF-κB signaling

The nuclear factor kappa-light-chain-enhancer of activated B cells (NF-κB) signaling pathway serves as a fundamental regulator of inducible gene expression within the immune system, modulating both innate and adaptive immune responses at multiple levels through its transcription factor family (55). This family comprises five distinct members: p65 (RELA), RELB, REL, NF-κB1 (p105/p50), and NF-κB2 (p100/p52) (71). Through its signaling cascade, NF-κB governs the transcription of numerous genes encoding proinflammatory cytokines and chemokines, thereby exerting a central role in shaping inflammatory processes (72).

The canonical NF-κB pathway is typically activated by molecular stimuli such as lipopolysaccharide (LPS), tumor necrosis factor-alpha (TNF-α), or interleukin-1 (IL-1), which engage Toll-like receptors (TLRs), TNF receptors (TNFRs), and IL-1 receptors (IL-1Rs), respectively (73). Ligand binding to these receptors initiates a cascade involving the IκB kinase (IKK) complex, which consists of the catalytic subunits IKKα (IKK1) and IKKβ (IKK2), as well as the regulatory component NEMO (also known as IKKγ). This complex phosphorylates the inhibitor proteins of κB (IκBs) that sequester the NF-κB dimers (typically p50 and p65) in the cytoplasm. Subsequent polyubiquitination and proteasomal degradation of IκBs liberate the NF-κB dimers, facilitating their nuclear translocation and transcriptional activation of target genes implicated in inflammatory and immune functions (71).

In addition to the canonical route, a non-canonical NF-κB signaling pathway exists, which is activated by a restricted group of ligands from the TNF superfamily and relies on the action of NF-κB-inducing kinase (NIK) (74). Collectively, NF-κB governs the expression of a diverse repertoire of genes, establishing complex regulatory circuits that involve cytokines, growth factors, cell adhesion molecules, intracellular mediators, transcriptional regulators, and microRNAs (miRNAs) (73).

### 5.2 NLRP3 lnflammasome and pyroptosis

Among the key molecular components implicated in the immunopathological complications of sepsis, the NLRP3 inflammasome has garnered significant attention due to its central role in the innate immune response (75, 76). Inflammasomes are cytosolic multiprotein platforms that facilitate the activation of caspase-1 and other inflammatory caspases, thereby amplifying inflammatory signaling. The NLRP3 inflammasome is composed of three principal elements: a sensor molecule (NLRP3), an adaptor protein (ASC—apoptosis-associated speck-like protein containing a caspase recruitment domain), and an effector protease (caspase-1) (77). Its functional activation is contingent upon a dual-signal mechanism: an initial priming phase, during which NLRP3 expression is upregulated via the NF-κB signaling axis, followed by a secondary activation signal that involves phosphorylation of ASC (76).

Upon assembly, the inflammasome facilitates the cleavage of pro-caspase-1 into its active form. This, in turn, catalyzes the maturation of the proinflammatory cytokines interleukin-1β (IL-1β) and interleukin-18 (IL-18), and initiates pyroptosis (78, 79) —a highly inflammatory form of programmed cell death mediated by gasdermin D. Specifically, caspase-1 cleaves gasdermin D, liberating its N-terminal fragment, which oligomerizes and forms pores in the plasma membrane, ultimately compromising cellular integrity (80) (**Figure 1**).

### 5.3 Reactive oxygen species and inflammatory feedback

Reactive oxygen species (ROS), including superoxide anions, hydrogen peroxide, peroxynitrite, hypochlorous acid, and hydroxyl radicals, are central mediators of the inflammatory response in sepsis. These molecules activate the NF-κB pathway, driving the production of key proinflammatory cytokines such as IL-1β, IL-6, and TNF-α. Concurrently, ROS enhance chemokine production and promote leukocyte recruitment to sites of inflammation, reinforcing the inflammatory milieu and establishing a self-sustaining cycle of immune activation and oxidative damage (1).

### 5.4 Therapeutic strategies targeting intracellular inflammatory signaling pathways

In recent years, increasing attention has been directed toward intracellular inflammatory signaling pathways as potential therapeutic targets in sepsis. Therapeutic approaches that target NF-κB signaling include IKK inhibitors, monoclonal antibodies, proteasome inhibitors, nuclear translocation inhibitors, DNA binding inhibitors, TKIs, non-coding RNAs, immunotherapy, and CAR-T (81).

Inhibitors of NF-κB, long considered a logical strategy to blunt cytokine-driven hyperinflammation, have shown stage-dependent effects in preclinical models. Early administration significantly reduced inflammatory injury and improved survival, while delayed treatment failed to provide benefit and in some cases worsened outcomes. This highlights the concept of a narrow therapeutic window for NF-κB inhibition, which likely explains the current lack of efficacy in phase II clinical trials. Another promising direction is represented by NLRP3 inflammasome antagonists, which aim to modulate both the hyperinflammatory response and subsequent immune dysfunction. DFV890 has undergone first-in-human evaluation and a phase 2a randomized trial in COVID-19 pneumonia, demonstrating safety, tolerability, and preliminary signals of clinical benefit. Similarly, dapansutrile (OLT1177), an oral selective NLRP3 inhibitor, has been tested in phase II studies for gout and phase Ib studies in heart failure, where it exhibited favorable safety and anti-inflammatory activity (82). Although these agents have not yet been validated in septic populations, their successful progression in other inflammatory conditions provides a strong rationale for future sepsis trials. Taken together, these findings indicate that immunotherapy in sepsis is evolving towards stage-specific and pathway-directed approaches, and that translational evidence from 2024–2025 has already started to reshape the therapeutic landscape (83).

## 6 Post-transcriptional regulation and intercellular communication

### 6.1 miRNAs and lncRNAs

A growing body of research underscores the regulatory role of long non-coding RNAs (lncRNAs) and microRNAs (miRNAs) in orchestrating the inflammatory milieu associated with sepsis (84). lncRNAs, defined as non-protein-coding transcripts exceeding 200 nucleotides in length (84), participate in a wide spectrum of cellular processes, including transcriptional modulation, alternative splicing, subcellular protein localization, cell cycle progression, proliferation, and programmed cell death (85).

miRNAs, in contrast, are shorter non-coding RNA molecules—typically 19 to 25 nucleotides—whose principal function lies in the post-transcriptional regulation of gene expression, predominantly via sequence-specific interactions with target mRNAs (86). Certain miRNAs, such as miR-494-3p and miR-218, act to attenuate inflammatory pathways by targeting receptors like Toll-like receptor 6 (TLR6), thereby dampening immune signaling cascades. Conversely, other miRNAs, including miR-21 and miR-208a-5p, are implicated in the modulation of inflammasome activation, apoptotic pathways, and myocardial injury, reflecting their multifaceted roles in sepsis-associated immune dysfunction (87).

Among the lncRNAs with immunoregulatory functions, lincRNA-Cox2 stands out for its capacity to influence immune gene transcription in both nuclear and cytoplasmic compartments through interactions with the NF-κB signaling axis. Similarly, lncRNAs such as lincRNA-EPS and lnc-13 exert repressive effects on immune gene expression, while Lethe and Lnc-DC modulate the activity of transcription factors NF-κB and STAT3, respectively.

Collectively, these classes of non-coding RNAs represent critical modulators of inflammation and are increasingly recognized as integral to the molecular pathogenesis of sepsis (85). Recent evidence highlights that both microRNAs and long non-coding RNAs have progressed beyond purely mechanistic observations and are under active evaluation as clinical biomarkers in sepsis. Circulating miR-150 and miR-223 are among the most consistently reported molecules associated with disease severity and mortality, and several meta-analyses demonstrate that panels combining multiple microRNAs improve prognostic accuracy compared with traditional scoring systems alone (88). Other candidates, such as miR-146a and miR-125b, are directly involved in the regulation of NF-κB activation and endotoxin tolerance and have been tested as dynamic markers of immune status. In parallel, lncRNAs such as NEAT1, MALAT1, and HOTAIR have been found to be upregulated in septic patients, with expression levels correlating with adverse outcomes (89, 90). These findings indicate that non-coding RNAs may not only serve as diagnostic and prognostic tools but could also guide patient stratification for personalized therapies. Nevertheless, the translation of these molecules into clinical practice is still hampered by the lack of standardized protocols for sample processing, heterogeneity among detection platforms, and the need for validation in multicenter, prospective cohorts (91).

### 6.2 Exosomes

Intercellular communication is a fundamental process in multicellular organisms, traditionally mediated through direct cell-to-cell contact or the secretion of soluble signaling molecules. In recent decades, extracellular vesicles (EVs), particularly exosomes, have emerged as a third modality of inter cellular communication (22). Exosomes are nanoscale vesicles, typically measuring between 40 and 160 nanometers in diameter (with a mean size of approximately 100 nm), that originate within multivesicular bodies (MVBs) and are released upon fusion of these organelles with the plasma membrane (92). The molecular composition of exosomes is highly context-dependent and reflects their cellular origin; they can transport a wide spectrum of biomolecules, including nucleic acids (DNA, various RNA species), lipids, metabolites, cytosolic and membrane-bound proteins, and immunologically active cytokines (93).

These vesicles exert profound immunomodulatory functions, shaping the immune microenvironment by influencing the phenotype and function of key effector cells such as polymorphonuclear neutrophils (PMNs), macrophages, dendritic cells (DCs), and lymphocyte subsets including T and B cells (92). In the context of sepsis-associated organ dysfunction, exosome-mediated signaling has emerged as a critical mechanism in the orchestration of disease progression (94). Proteomic profiling of exosomes derived from septic animal models has demonstrated an early elevation of proinflammatory cytokines, including IL-1β, IL-2, IL-6, and TNF-α, followed by increased expression of additional cytokines such as IL-12, IL-15, IL-17, and IFN-γ. This temporal pattern is consistent with the biphasic immune trajectory of sepsis, wherein the later stages are marked by a compensatory rise in anti-inflammatory mediators such as IL-4 and IL-10 (95).

The release of inflammatory exosomes during sepsis has been linked to the activation of Toll-like receptors (TLRs), particularly in response to microbial components such as lipopolysaccharides (LPS) or whole pathogens (95). These exosomes, derived from septic organisms or LPS-stimulated cells, have the capacity to further activate TLR pathways—most notably TLR2, TLR4, and TLR7—within recipient immune cells such as macrophages (96). This engagement precipitates activation of the NF-κB signaling axis, thereby enhancing the transcription of key proinflammatory cytokines and chemokines, including IL-6, TNF-α, and IL-1β (95). Additionally, the pronounced increase in circulating exosomes observed in sepsis correlates with elevated levels of damage-associated molecular patterns (DAMPs), suggesting that exosomes may function as critical vectors for systemic DAMP dissemination and thus represent central agents in the propagation of sepsis pathophysiology (22). Recent clinical and translational studies have underscored the role of exosomes beyond mechanistic insights, highlighting their potential as diagnostic and prognostic biomarkers in sepsis. Circulating exosomal microRNAs have been shown to predict disease severity and mortality, with panels including miR-21, miR-146a, and let-7 family members showing strong discriminative capacity for septic shock and poor outcomes (97). In particular, exosomal miRNA signatures have outperformed conventional markers such as C-reactive protein and procalcitonin in differentiating sepsis from non-infectious systemic inflammation (98). Moreover, organ-specific exosomes may provide early indicators of sepsis-associated organ dysfunction: renal exosomes enriched in NGAL or KIM-1 reflect tubular injury, while cardiac exosomes containing miR-21 correlate with septic cardiomyopathy (99). Beyond their role as biomarkers, exosomes are increasingly investigated as therapeutic agents or delivery vehicles. Mesenchymal stem cell-derived exosomes, for example, exhibit immunomodulatory effects by attenuating NF-κB activation, reducing cytokine storm severity, and promoting tissue repair in preclinical sepsis models (100). These findings suggest that exosomes may serve as minimally invasive tools for patient stratification, as well as innovative therapeutic platforms, although standardization of isolation techniques and validation in multicenter trials remain essential for clinical translation.

## 7 Neutrophil extracellular traps

### 7.1 NET formation mechanisms

Neutrophil extracellular traps (NETs) are web-like structures released by activated neutrophils into the extracellular milieu. Composed of decondensed chromatin, histones, nucleosomes, and proteolytic enzymes such as neutrophil elastase (NE), myeloperoxidase (MPO), and cathepsin G, these complexes represent an essential component of the innate immune arsenal (5, 22, 101). The constituents of NETs act synergistically to immobilize and neutralize a broad array of microbial pathogens, including Gram-positive and Gram-negative bacteria, viruses, fungi, protozoa, and other parasites that are otherwise capable of evading phagocytic clearance. The formation of NETs entails the fusion of nuclear DNA and chromatin with antimicrobial molecules derived from neutrophil granules, followed by the ejection of this composite material into the extracellular environment, where it assembles into filamentous structures with potent antimicrobial activity (5, 26, 101).

Initial experimental evidence demonstrated that NET release could be triggered by neutrophil exposure to activators such as phorbol 12-myristate 13-acetate (PMA), interleukin-8 (IL-8), and bacterial lipopolysaccharide (LPS). Subsequent investigations have broadened the spectrum of NET-inducing agents to encompass various microbial and non-microbial stimuli, including bacteria, viruses, fungi, yeast, parasites, and the plant lectin concanavalin A (22). Furthermore, NETs components, including neutrophil elastase, myeloperoxidase, histones, and matrix metalloproteinases, retain direct cytotoxic activity which further contributes to the clinical deterioration of sepsis (102).

### 7.2 Dual role in host defense and tissue injury

In the context of sepsis, dysregulated NET formation, particularly when accompanied by inadequate mechanisms for their clearance, has been implicated in exacerbating the pathophysiological trajectory of the disease (101). Three mechanistically distinct forms of NET release, collectively referred to as NETosis, have been delineated: suicidal NETosis, vital NETosis, and mitochondrial NETosis. In the suicidal variant, NET formation culminates in neutrophil lysis and cell death (103), a process that may contribute to the immunosuppressive state frequently observed in the later phases of sepsis (101). However, the functional significance of pathogen-induced suicidal NETosis remains debated, with ongoing discourse as to whether it constitutes a deliberate antimicrobial defense or represents a maladaptive immune response facilitating pathogen persistence (104).

Extracellular histones associated with NETs function as damage-associated molecular patterns (DAMPs), activating Toll-like receptors (TLRs) and initiating downstream inflammatory signaling cascades that amplify the systemic inflammatory response typical of sepsis (105). Moreover, NETs influence macrophage polarization, promoting a proinflammatory M1-like phenotype characterized by the secretion of cytokines such as IL-1, IL-6, IL-8, and tumor necrosis factor (TNF) (11).

The interplay between neutrophils and endothelial cells further augments NET formation, a process partially mediated by IL-8 released by activated endothelial cells (22). The histone components of NETs exhibit pronounced cytotoxicity toward endothelial cells, and the ensuing endothelial damage constitutes a critical factor in the pathogenesis of multiorgan dysfunction syndrome (MODS) during sepsis (104).

## 8 Complement system

### 8.1 Key components and activation

The complement system comprises an intricate network of more than 50 proteins, many of which exist in inactive precursor forms (zymogens) and require proteolytic cleavage for activation (80). Dysregulated complement activation has been consistently associated with elevated mortality rates in patients with sepsis. During septic progression, the complement cascade and cytokine networks are often co-activated, and their respective effector molecules frequently exhibit overlapping biological functions (106).

In the early phases of the hyperinflammatory response, elevated levels of activated complement fragments—particularly C3a, C4a, and C5a, which function as potent proinflammatory anaphylatoxins—act to intensify the systemic inflammatory milieu. Among these, C5a plays a particularly pivotal role in directing neutrophil migration to sites of infection and tissue injury (5).

#### 8.1.1 Contribution to immunosuppression

Additionally, experimental models, such as cecal ligation and puncture (CLP) in rodents, have demonstrated extensive apoptosis within the thymus during sepsis. This process appears to be mediated by the interaction between C5a and its receptor, C5aR, and has been implicated in the pathogenesis of sepsis-induced immunosuppression (107).

## 9 Endothelial activation

### 9.1 Response to PAMPs, NETs, and cytokines

Due to their strategic anatomical position at the interface between blood and tissues, endothelial cells (ECs) serve as primary immunological sentinels, rapidly responding to the presence of circulating microbial agents. In the context of sepsis, ECs are among the earliest cellular targets activated by pathogen-associated molecular patterns (PAMPs), neutrophil extracellular traps (NETs), and a range of proinflammatory cytokines, including tumor necrosis factor-alpha (TNF-α), interleukin-6 (IL-6), and interleukin-1 (IL-1) (12).

Endothelial cells express a repertoire of Toll-like receptors (TLRs), which play a pivotal role in pathogen recognition and immune activation (108). Notably, the expression of TLR2 and TLR4 on ECs can be modulated in response to infection and inflammatory stimuli, either directly through exposure to lipopolysaccharide (LPS) or indirectly through cytokines such as TNF-α and interferon-gamma (IFN-γ) (109).

#### 9.1.1 Pro-thrombotic and inflammatory roles

The activation of TLRs, as well as stimulation by NETs—primarily through the NF-κB signaling pathway—elicits proinflammatory and pro-thrombotic responses in endothelial cells, activating platelets and stimulating the deposition of fibrin, thereby contributing to the amplification of vascular inflammation, blood clot formation and the overall pathogenesis of sepsis (11, 12, 110).

## 10 Multiple organ dysfunction syndrome

### 10.1 Oxidative stress and mitochondrial dysfunction

In the context of sepsis, a significant elevation in oxidative stress arises primarily from the excessive generation of reactive oxygen species (ROS), a process largely mediated by proinflammatory cytokines such as interleukin-6 (IL-6), interleukin-1 (IL-1), and tumor necrosis factor-alpha (TNF-α) (55). This oxidative burden contributes to mitochondrial dysfunction, resulting in cytopathic hypoxia, impaired bioenergetic processes, and activation of apoptotic pathways, ultimately culminating in tissue injury and organ failure (1). Specifically, mitochondrial oxidative phosphorylation (OXPHOS) is hindered by either hypoxic conditions or ROS-induced structural damage, leading to a pronounced reduction in adenosine triphosphate (ATP) production. To compensate for this energy deficit, affected cells undergo metabolic reprogramming, shifting preferentially toward glycolysis as the dominant energy-generating pathway (111).

**Figure 2:**
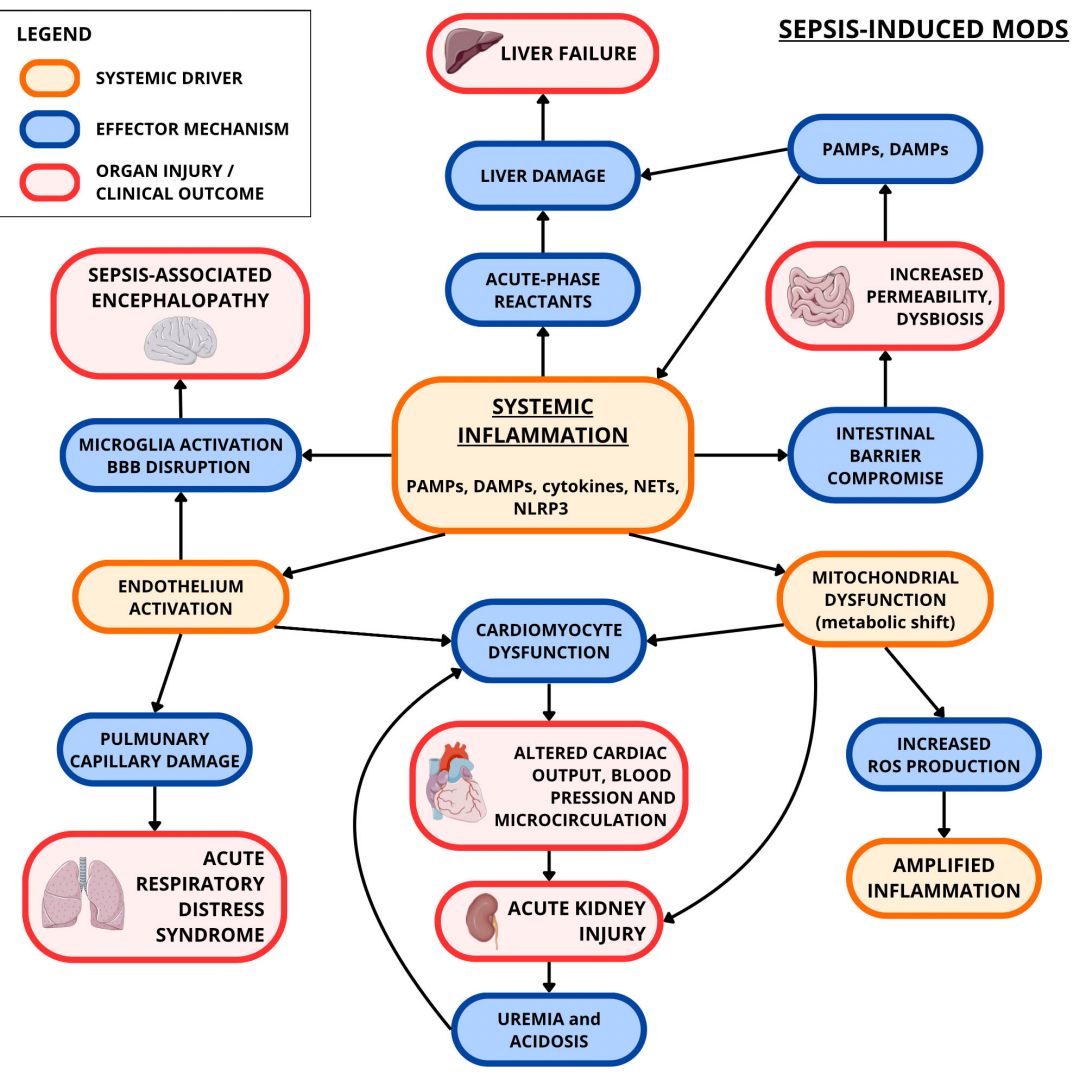
Sepsis-induced MODS (focus on heart, kidneys, brain, liver, gut, and lungs). Systemic inflammation, determined by the interplay of PAMPs, DAMPs, and cytokines, contributes to the production of more cytokines and ROS. This oxidative stress, along with the inflammation itself, contributes to mitochondrial dysfunction which results in further ROS production and insufficient cellular energy metabolism. Meanwhile, inflammation causes endothelial activation and damage. These two conditions have harmful consequences on the myocardium, renal nephrons, pulmonary circulation, intestinal barrier, blood-brain barrier (BBB), and liver. This may result respectively in cardiomyocyte dysfunction, acute kidney injury (exacerbated by cardiomyocyte dysfunction’s effects), acute respiratory distress syndrome (ARDS), dysbiosis and increased intestinal permeability (with more PAMPs and DAMPs going to the liver), sepsis-associated encephalopathy (exacerbated by inflammation-induced microglial activation), and liver failure. The artwork used in this figure was adapted from Servier Medical Art (https://smart.servier.com/). Servier Medical Art by Servier is licensed under CC BY 4.0 (https://creativecommons.org/licenses/by/4.0/).

However, this adaptive shift is metabolically inefficient and insufficient to sustain normal cellular function, thereby aggravating cellular stress and contributing to progressive tissue damage. When widespread, this dysregulated metabolic response evolves into a systemic process, promoting the onset of multiple organ dysfunction syndrome (MODS), sustained by intricate inter-organ communication networks (112, 113) (**Figure 2**).

A critical contributor to this pathological state is endothelial dysfunction, which impairs the endothelium’s ability to regulate vascular tone, barrier integrity, and permeability. The resultant capillary leakage facilitates fluid extravasation into interstitial compartments, producing edema and compromising tissue perfusion (13).

### 10.2 Organ-specific involvement

Among the organs most vulnerable to MODS-related cross-talk are the heart, lungs, kidneys, liver, gastrointestinal tract, and brain (113).

#### 10.2.1 Heart involvement

Myocardial dysfunction, frequently observed in sepsis, is a major prognostic factor associated with increased mortality (114). Systemic inflammation triggers excessive catecholamine release and perturbs myocardial contractility, further impairing cardiac output. These alterations are exacerbated by the previously discussed metabolic reprogramming, which diminishes the energetic capacity of cardiomyocytes (115). It has been proposed that inflammatory mediators—including TNF-α, IL-1, IL-6—as well as bacterial toxins such as endotoxins and exotoxins, exert direct cytotoxic effects on cardiac tissue, thereby impairing cardiomyocyte viability and function (116). Additionally, coronary endothelial cells increase the expression of vascular cell adhesion molecule-1 (VCAM-1), promoting neutrophilic infiltration into myocardial tissue (117). The role of heart-derived cytokines (cardiokines) in mediating bidirectional communication between the cardiovascular system and distant organs has also gained increasing recognition in the pathophysiology of sepsis (113).

#### 10.2.2 Kidneys involvement and cardiorenal syndrome

Renal involvement in sepsis is typified by microcirculatory abnormalities, hypoperfusion secondary to hypovolemia and hypotension, ischemia-reperfusion injury, and Toll-like receptor (TLR)-mediated activation in tubular epithelial cells. These mechanisms enhance oxidative stress at the renal level and contribute to the development of acute kidney injury (AKI). Proinflammatory cytokines, in concert with activated leukocytes that may obstruct the capillary microcirculation and promote microthrombosis, further compromise renal function. In response to this stress, nephrons adapt by prioritizing cellular survival mechanisms at the expense of their physiological roles in filtration and reabsorption (13, 118). A clinically relevant consequence of this interaction is the emergence of type 5 cardiorenal syndrome (CRS), in which myocardial and renal dysfunction coexist and exacerbate each other through shared pathophysiological mechanisms. Factors such as uremia, metabolic acidosis, ischemia, and abnormal cardiac output contribute to this vicious cycle, with cytokines—particularly TNF-α—implicated in its progression. However, the full mechanistic pathways remain incompletely understood (119). Recent mechanistic studies have clarified the role of inflammatory cytokines in mediating bidirectional heart–kidney dysfunction during sepsis. Tumor necrosis factor-α (TNF-α) is a central mediator of this process. In cardiomyocytes, TNF-α induces nitric oxide synthase and enhances the generation of reactive oxygen species, leading to impaired calcium handling and depressed contractility, which are hallmarks of septic cardiomyopathy. At the renal level, TNF-α activates NF-κB and p38 MAPK pathways in microvascular endothelial cells, causing increased permeability, loss of barrier function, and microvascular thrombosis. These effects reduce renal perfusion and worsen acute kidney injury. Together, these cytokine-driven alterations establish a feed-forward loop in which myocardial depression and renal dysfunction amplify one another in a vicious cycle, providing a molecular explanation for the pathophysiology of type 5 cardiorenal syndrome in sepsis (120, 121).

#### 10.2.3 Lungs and acute distress respiratory syndrome

The lungs represent one of the most frequently and severely affected organs in sepsis. Patients often develop acute lung injury (ALI), which, if left unchecked, may progress to acute respiratory distress syndrome (ARDS) (122). Inflammatory damage to pulmonary capillary endothelium activates local immune cells, amplifying the inflammatory milieu within the pulmonary microenvironment. This cascade results in epithelial and endothelial injury, disruption of barrier function, enhanced oxidative stress, and edema—hallmarks of ARDS pathogenesis (123).

#### 10.2.4 Brain and septic encephalopathy

Sepsis-associated encephalopathy (SAE) constitutes another severe complication, with early manifestations including delirium, cognitive disturbances, and emotional dysregulation, potentially progressing to coma in advanced stages (124). Disruption of the blood-brain barrier (BBB) permits the influx of inflammatory and neurotoxic mediators into the central nervous system (125). Within the cerebral milieu, both pro- and anti-inflammatory cytokines—such as TNF-α, IL-1, TGF-β, and monocyte chemoattractant protein-1 (MCP-1)—may contribute to neuronal dysfunction, potentially through interactions with NMDA receptors and other critical signaling pathways. Microglial activation and cerebral endothelial dysfunction further intensify this neuroinflammatory response (125, 126).

#### 10.2.5 Liver involvement

The liver, a central organ in host defense, metabolic regulation, and coagulation, exhibits a paradoxical role in sepsis. While its immune activity is essential for neutralizing pathogens and endotoxins, it may simultaneously amplify systemic inflammation and exacerbate injury in distant organs (127). Hepatic involvement in sepsis spans from subclinical biochemical abnormalities to overt clinical syndromes such as jaundice, cholestasis, or ischemic hepatitis. IL-6 is a key modulator of the hepatic inflammatory response, inducing the synthesis of acute-phase reactants including C-reactive protein (CRP), α1-antitrypsin, fibrinogen, prothrombin, and haptoglobin. Expression of IL-6 is itself upregulated in response to both endotoxins like lipopolysaccharide (LPS) and other proinflammatory stimuli, including TNF-α (128). The progression of hepatic dysfunction ranges from reversible metabolic alterations to irreversible structural damage and liver failure (127).

#### 10.2.6 Gastrointestinal tract and gut-liver axis

Finally, the gastrointestinal tract is also significantly affected in sepsis. Mucosal injury and epithelial apoptosis disrupt the intestinal barrier, increasing permeability and facilitating microbial translocation. These alterations are mediated, in part, by TLR4 activation, which also contributes to the depletion of intestinal stem cells. This breach of mucosal integrity leads to gut dysbiosis and facilitates the translocation of PAMPs and DAMPs into the portal and biliary circulations. The resultant hepatic inflammatory response underscores the critical role of the gut-liver axis in the systemic pathogenesis of sepsis (129). Mechanistically, microbial translocation in sepsis results in the activation of hepatic Kupffer cells through TLR4 signaling, which triggers downstream NF-κB activation and induces the release of proinflammatory cytokines such as TNF-α and IL-1β. This cascade amplifies leukocyte recruitment, endothelial activation, and local hepatocellular injury. In parallel, cytokine-driven cross-talk between Kupffer cells, hepatic stellate cells, and infiltrating neutrophils perpetuates liver inflammation and microvascular dysfunction. Beyond the acute inflammatory burst, the persistent influx of PAMPs and DAMPs sustains an altered immunological environment, contributing to cholestasis and impaired hepatic clearance of endotoxins. These molecular pathways illustrate that sepsis-associated liver dysfunction is not simply secondary to hypoperfusion but is directly linked to cytokine-mediated immune activation originating from the gut (130).

## 11 Discussion

The growing body of experimental and clinical data has delineated an increasingly nuanced view of sepsis as a dynamic syndrome, in which the balance between proinflammatory and immunosuppressive responses plays a central role in determining prognosis (3, 9). It is now widely accepted that sepsis does not result from a single linear cascade of events, but rather from a complex interplay between immunologic, metabolic, and molecular signals, which are modulated in time and space (8, 17).

However, this complexity has generated different interpretative models, with some authors arguing for sequential phases— hyperinflammatory followed by immunosuppression — while others describe a functional overlap from the first hours of the septic event (9, 31, 32, 101). This dualism is not purely theoretical but has significant clinical implications, particularly when determining the optimal timing for immunomodulatory interventions (8–10).

**Figure 3:**
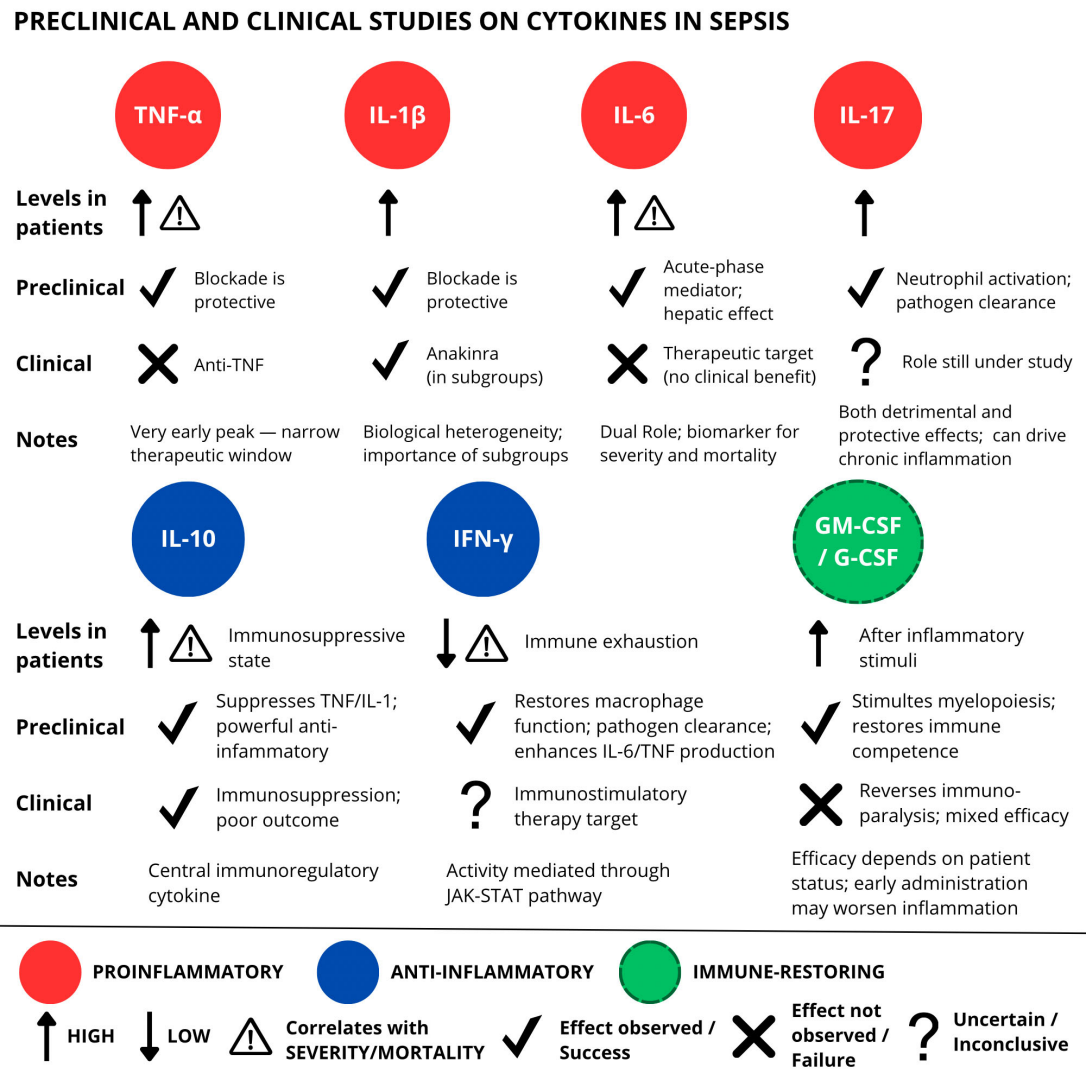
Overview of preclinical and clinical studies on cytokine-targeted therapies in sepsis. While preclinical models demonstrated encouraging effects of cytokine modulation, clinical trials have yielded inconsistent or limited results, emphasizing the translational challenges in this field.

The reviewed literature confirmed the central role of the so-called cytokines storm in activating the systemic inflammatory cascade (5) (**Table 2**, **Figure 3**). In this scenario, proinflammatory cytokines, such as TNF-α, IL-1β, and IL-6, have been confirmed to act in the acute phase of sepsis (32), mediating the systemic response and progression to multiorgan dysfunction (9, 34, 47). Conversely, alternative arguments have arisen concerning the involvement of other cytokines. Some authors have pointed out the involvement of cytokines traditionally considered protective and anti-inflammatory, such as TGF-β, in immune dysfunction. This involvement may contribute to persistent immunosuppression (32) and promote the expansion of regulatory T cells or the differentiation of proinflammatory lymphocyte subpopulations, such as Th17 (59, 60).

**Table 2:** Summary of preclinical and clinical studies on cytokines in sepsis (**Figure 3**).

Cytokine	Preclinical evidence (animal studies)	Clinical evidence (human studies)	Key references
TNF-α	Rapid rise post-LPS in mice; key driver of SIRS and organ failure; knockout or neutralization improves survival.	Elevated in septic patients; correlated with mortality; anti-TNF trials inconclusive.	Preclinical: Bhatia M (2009) Clinical: Wautier et al. (2023); wang et al. (112)
IL-1β	Potent proinflammatory cytokine; induces fever and organ dysfunction; IL-1RA (e.g. Anakinra) improves outcomes in models.	Elevated in septic patients; Anakinra shows promise in selected patient subgroups.	Preclinical/Clinical: Shimabukuro-Vornhagen et al. (2018)
IL-6	Induced by TNF/IL-1; dual role via classical vs. trans-signaling; pivotal in hepatic acute-phase response.	Biomarker for severity and mortality; clinical targeting explored more in COVID-19 than sepsis.	Preclinical: Jarczak & Nierhaus (2022) Clinical: Liu et al. (2022)
IL-10	Anti-inflammatory cytokine; suppresses TNF, IL-1; knockout mice show exaggerated inflammation.	High IL-10 correlates with immunosuppression and poor outcome in late sepsis.	Clinical: Liu et al. (2022)
IFN-γ	Activates macrophages; IFNγR1 deficiency increases infection risk in mice; enhances IL-6, TNF-α production.	Low IFN-γ in late sepsis correlates with immune exhaustion; possible marker for immunostimulatory therapy.	Preclinical: Ding et al. (2022) Clinical: Carcillo & Shakoory (2024)
IL-17	Recruits neutrophils; supports pathogen clearance in early infection but can drive chronic inflammation.	Role still under study; both protective and detrimental effects noted in sepsis context.	Preclinical: Chousterman et al. (2017)
GM-CSF/G-CSF	Stimulates myelopoiesis; restores immune competence in animal models; early administration may worsen inflammation.	Studied in trials to reverse immunoparalysis; mixed efficacy depending on patient status.	Preclinical/Clinical: Mathias et al. (2015)

In addition, the ambivalent role of other cytokines further complicates the identification of therapeutic targets. IL-6, for example, exerts both proinflammatory effects via the membrane receptor IL-6R and anti-inflammatory effects via trans-signaling; and IL-8 can both promote or inhibit the production of pro-inflammatory cytokines depending on the specific context (32).

These dualities underscore the complexity of cytokine interactions in the context of sepsis, highlighting the importance of a phenotypic and dynamic approach to this complex disease (5, 131). In this context, therapeutic intervention should be guided by clinical and temporal context rather than static assumptions (8–10).

In parallel, a growing interest has emerged towards mediators such as miRNAs, lncRNAs (85, 132), exosomes (94), and NETs, which appear to profoundly influence the immune-metabolic axis (101), despite their clinical application remains to be fully explored (133). Some miRNAs and lncRNAs, such as miR-494-3p and miR-218, play a significant role in reducing inflammatory activity and cell polarization (85, 132) by inhibiting receptors, including TLR6, and interacting with signaling pathways, such as NF-κB and STAT3. Others, such as miR-21 and miR-208a-5p, are involved in modulation of the inflammasome, apoptosis, and myocardial damage, highlighting diverse roles of these mediators in the immune dysfunction of sepsis (87).

Prominent among lncRNAs is lincRNA-Cox2, which regulates transcription of immune genes at both the nuclear and cytoplasmic levels through interaction with NF-κB (134). Other lncRNAs, such as lincRNA-EPS and lnc-13, repress the expression of immune genes, while Lethe and Lnc-DC modulate the activities of NF-κB and STAT3, respectively. Altogether, these noncoding RNAs are considered key modulators of inflammation in sepsis (85).

Similarly, exosomes released by immune and tissue cells are increasingly recognized as vectors of immunological information that can amplify or modulate the systemic response (22, 94). During sepsis, exosomes contribute to the progression of inflammation by carrying proinflammatory cytokines (such as IL-1β, IL-6, TNF-α) and activating Toll-like receptors (TLR2, TLR4, TLR7) in immune cells. This stimulates the NF-κB pathway, amplifying the inflammatory response. In more advanced stages, exosomes also carry anti-inflammatory cytokines (IL-4, IL-10), reflecting the biphasic nature of the immune response in sepsis (95).

An additional level of complexity in the pathophysiology of sepsis involves the intracellular mechanisms regulating inflammation. The transcription factor NF-κB represents a central node in the immune response, as it is typically activated by molecular stimuli such as lipopolysaccharide (LPS), tumor necrosis factor-alpha (TNF-α), or interleukin-1 (IL-1), which engage Toll-like receptors (TLRs), TNF receptors (TNFRs), and IL-1 receptors (IL-1Rs), respectively. NF-κB is capable of inducing the expression of numerous proinflammatory cytokines, chemokines, and adhesion molecules (72, 73). In close connection with NF-κB, the NLRP3 inflammasome constitutes another critical element of the inflammatory response. Its activation requires a priming signal-mediated precisely by NF-κB (75, 76), leading to caspase-1 activation, secretion of IL-1β and IL-18, and initiation of pyroptosis (78, 79). Although its protective function is evident in the early response against pathogens, the extent to which persistence or dysregulation of inflammasome activity may contribute to immune dysfunction or tissue damage in advanced sepsis remains to be elucidated (7, 79). For example, in this context, pyroptotic cardiomyocyte death represents a key mechanism underlying sepsis-induced myocardial dysfunction (SIMD), a complication associated with poor outcomes and increased mortality (76).

NETs represent another nuanced area of research. While they contribute to neutralization of pathogens through physical and biochemical mechanisms (5, 22), they have also been associated with pro-thrombotic effects, endothelial activation, and propagation of organ damage (101). Their role thus appears to be twofold and highly dependent on the temporal context and the body’s ability to dispose of them effectively (104).

In sepsis, the dysregulated formation of NETs, especially if not adequately removed, contributes to the aggravation of the disease. The interaction between neutrophils and endothelial cells amplifies their production, in part mediated by IL-8 released by activated endothelium (22, 101, 110). Extracellular histones associated with NETs function as DAMPs activating TLRs and initiating downstream inflammatory signaling cascades that amplify the sepsis systemic inflammatory response, and the resulting damage is a key element in the development of MODS (105). In addition, NETs influence macrophage polarization, promoting a proinflammatory M1 phenotype associated with the secretion of cytokines such as IL-1, IL-6, IL-8, and TNF (11).

The complement system, critical in antimicrobial defense, also shows ambivalent functional characteristics. Activation of its three main pathways leads to the generation of mediators such as C3a and C5a, which promote leukocyte recruitment and cytokine production (106). However, excess C5a has been associated with extensive thymus apoptosis in CLP in rodents, it may be involved in sepsis-associated immunosuppression pathogenesis. These results highlight that complement may contribute as much to the protection as to the pathogenesis of sepsis in a time-dependent manner (107).

Activation of vascular endothelium contributes to the pathogenesis of sepsis. ECs are among the earliest cellular targets activated by PAMPs, NETs, and a range of proinflammatory cytokines, including TNF-α, IL-6, and IL-1 (11, 12, 110). And, in response to cytokines such as TNF-α and IL-1β, can express adhesion molecules (ICAM-1, VCAM-1, E-selectin) that facilitate leukocyte recruitment (118). This is associated with increased permeability, fluid extravasation, activation of coagulation, and interaction with components of complement and NETs (12, 13).

Equally controversial is the study of metabolic changes observed in sepsis. The link between inflammation and cellular metabolism emerges strongly, particularly in the description of the transition from oxidative to glycolytic metabolism that occurs in both immune cells and peripheral tissues to compensate for the pronounced reduction in ATP production connected to mitochondrial structural damages caused by a ROS increase mediated by inflammatory signals, and hypoxic conditions (55, 111). Such metabolic reorganization is interpreted by some as an adaptive mechanism, by others as an expression of irreversible energetic dysfunction that may contribute to progressive tissue damage and the onset of MODS if protracted over time (111–113).

The terminal phase of sepsis frequently manifests with the appearance of multiorgan dysfunction (MODS), a clinical outcome of an uncontrolled systemic inflammatory response and large-scale molecular damage. The simultaneous or progressive involvement of several organs reflects the interaction between immune mediators, oxidative stress, metabolic alterations, and endothelial damage, in a cascading dysregulation framework. The literature reviewed highlights how ROS, proinflammatory cytokines, and products of NETosis are capable of directly damage key cellular structures, including DNA, mitochondria, and membrane proteins (13, 112, 113).

The lung is one of the most frequently affected organs in sepsis, with development of ARDS induced by increased vascular permeability, accumulation of cellular infiltrates, and endothelial activation (122, 123). The kidney undergoes acute tubular damage, influenced by both hypoperfusion and systemic and local inflammatory signals (13, 118). The heart, despite the absence of direct ischemic damage, shows reduced contractility due to cytotoxic effects of cytokines (including TNF-α, IL-1, IL-6), bacterial toxins and ROS on cardiac tissues. In addition, ECs activation leads to neutrophilic infiltration into myocardial tissue (113, 116, 117). Myocardial and renal dysfunction can exacerbate each other in a condition known as type 5 cardiorenal syndrome (119). The intestine and liver are equally affected: the former through epithelial barrier dysfunction and bacterial translocation, the latter with cholestasis and hepatocyte necrosis. While the liver’s is essential for neutralizing pathogens and endotoxins, it may simultaneously amplify systemic inflammation exacerbating injury in distant organs, showing therefore a paradoxical role (127–129). Finally, the central nervous system may undergo alterations in blood flow, blood-brain barrier integrity, and synaptic transmission, contributing to the clinical picture of septic encephalopathy (125, 126).

These observations underscore how organ dysfunction represents not only the passive result of systemic inflammation but also the outcome of a vicious cycle sustained by the complex interactions between the immune, metabolic, vascular, and nervous systems (112, 113). Future research should focus on strategies that are not limited to suppressing the inflammatory response, but rather on preserving or restoring cell and tissue function in the various organs involved.

Thus, despite the relevant disagreements emerged from the analysis of the literature, there is consensus on a few key aspects: sepsis is a complex syndrome and the result of a disregulated immune response in which different immune and metabolic mediators interact, it actively involves transcriptional and post-transcriptional regulation. It is characterized by aberrant communication between organs and systems. Mitochondrial dysfunction, oxidative stress, and loss of endothelial integrity are common events in various organs, contributing to the development of MODS.

The current challenge no longer lies in identifying individual mediators but rather in understanding the functional interactions between proinflammatory, anti-inflammatory, regulatory, and metabolic pathways, with the goal of guiding future therapeutic approaches, tailored to the immunologic profile and specific time of disease (3, 131, 135, 136). The contemporary approach to managing sepsis focuses on stabilizing hemodynamics and controlling the underlying infection (137). The repeated failure of cytokine-targeted therapies such as anti-TNF, anti-IL-1, and anti-IL-6 antibodies in sepsis reflects systemic issues in trial design and patient selection rather than the absence of biologic relevance. Most studies enrolled highly heterogeneous populations, encompassing both hyperinflammatory and profoundly immunosuppressed patients, thereby diluting potential benefits. In addition, interventions were often administered too late, missing the narrow window when cytokine blockade may be effective. The redundancy of cytokine signaling further complicates this approach, as inhibition of a single mediator can be compensated by parallel pathways. Future strategies must therefore move beyond the “one-size-fits-all” paradigm and embrace personalized immunotherapy. This requires stratifying patients according to immunological endotypes defined by biomarkers such as monocyte HLA-DR expression, soluble urokinase plasminogen activator receptor, ferritin, or transcriptomic signatures. Adaptive clinical trial designs that target hyperinflammatory or immunoparalysis phenotypes separately may allow more precise interventions. Illustrative examples already exist: anakinra has shown survival benefit in septic patients with macrophage activation syndrome-like features, while interferon-γ has improved immune recovery in patients with sepsis-associated immunoparalysis. These experiences demonstrate that the failures of past cytokine-directed therapies stem from a mismatch between patient, target, and timing, and strongly support a transition towards biomarker-guided, individualized treatment strategies (137, 138).

A more comprehensive understanding of the cellular and molecular elements involved in the host response to infection can help the decision-making process, especially in resource-limited environments where standard guidelines may be less applicable (139). The in-depth study of novel mediators, such as miRNAs, lncRNAs, and exosomes, as well as immune-metabolism or inter-organ interactions, opens perspectives relevant to the identification of new biomarkers and therapeutic targets in sepsis. Emerging evidence suggests that selective immunomodulation, targeting specific cytokines or intracellular pathways such as NF-κB, may be an effective therapeutic strategy, provided it is guided by a temporal and functional characterization of each patient’s immune status (17, 97, 133). Research perspectives point toward an integrated and personalized approach, in which the immunologic and metabolic phenotyping of the patient can guide targeted interventions, adaptable to the different stages of the disease, fostering the development of predictive models and integrated therapeutic approaches that can consider the biphasic and adaptive nature of the human immune system under critical conditions (14, 131, 135, 140).
